# Fractal analysis of the microstructure of milk powders produced at various temperatures

**DOI:** 10.1007/s13197-020-04268-x

**Published:** 2020-02-11

**Authors:** Michał Smoczyński

**Affiliations:** Department of Dairy Science and Quality Management, The Faculty of Food Sciences, University of Warmia and Mazury, Oczapowskiego 7, 10-719 Olsztyn, Poland

**Keywords:** Milk powder, Image analysis, Fractal dimension, Particle morphology

## Abstract

The quality of milk powder is largely determined during it manufacture process by the morphological characteristics of powder particles. Considering that, the main research objective of this study was to determine whether parameters of the production process contribute to differences in the microstructure of the manufactured powders, and by this means affect their functional traits. To diversify the milk powder production process, various temperatures of the inlet air were used during drying, i.e. 140, 150 and 160 °C. An image fractal analysis was employed and powder particle sizes were compared with respective results achieved in the instrumental analysis using the laser diffraction method. Values of fractal dimensions decreased slightly along with drying temperature increase, what demonstrates that the technological parameters are reflected in the microstructure of milk powders produced. Although particle sizes determined with both methods fitted within the same range of values, the contribution of particular fractions slightly differed and the choice of the appropriate approach may not always be unambiguous. Finally, the fractal dimension is a precise parameter which provides the accurate and explicit characteristics of milk powder microstructure and as such should be recommended for the characterization of irregular structures of different food products.

## Introduction

Milk powder production is an important branch of the dairy industry. Milk powder has multiple advantages and represents a stable, easy to store and transport product. The drying of milk ensures preservation of its stability in time by eliminating bacteria growth and by reducing the kinetics of food powder components degradation (Fitzpatrick and Ahrne 2005). Milk powder is additionally easy and convenient to use, hence it is widely utilized as an additive in many products in the food industry.

Apart from the chemical composition, particle morphology is deemed to be one of the key traits which affect the functional characteristics of milk powders. Particle size, particle size distribution, and particle shape determine the desired functional properties and, consequently, the quality of milk powders. Therefore, the examination of properties of milk powder particles is important while predicting and controlling properties and quality of milk powders in the course of their production process as well as during their subsequent storage or transport. Microscopy has been found a useful tool in analyzing their microstructure (Burgain et al. 2017; Ho et al. 2017; Kalab et al. 1995). However, even though the microscopic images provide multiple valuable data, the unbiased comparison of similar structures with this method is often difficult. A solution in this case is offered by image analysis, which enables employing mathematical tools for explicit evaluation and comparison of structures examined. In the case of non-uniform and irregular structures—like e.g. milk powder particles—useful appears also the concept of fractals and fractal dimension (Mandelbrot 1977). The fractals allow finding the order in seemingly chaotic and unordered structures or phenomena. In the classic Euclidean geometry, dimensions of a point, a line, a surface, and a space account for 0, 1, 2, and 3, respectively, whereas in the case of fractal objects their dimensions may attain intermediate values between these mentioned above (Barrett and Peleg 1995). In the case of the analysis of a two-dimensional image of milk powder, this dimension may attain the value between 1 and 2, thus indicating the extent of irregularity (surface filling) of particle contour.

The main objective of this study was to verify a research hypothesis whether various parameters of the production process contribute to differences in the microstructure of manufactured powders, thereby affecting their functional traits. To this end, particles of milk powders produced via spray drying at various temperatures were subjected to an image analysis coupled with fractal dimension measurement using the area perimeter method (Dziuba et al. 1999). Particle size was additionally determined with the laser diffraction method. Afterwards, results of particle size analyses were compared with these of image analysis to verify the reliability of the fractal model in morphological analyses of the particles examined.

## Material and methods

Milk samples were collected from a Technological Hall of the Department of Dairy Industry and Quality Management, University of Warmia and Mazury in Olsztyn, which is equipped in complete dairy production lines. Raw milk was purchased by the Department from dairy production plants operating in the north-eastern Poland. Milk was pasteurized (72 °C/30 s), heated to 40 °C, and centrifuged to obtain two fractions: skimmed milk and sweet cream. The skim milk was analyzed for its chemical composition with a MilkoScan FT2 apparatus and processed further to obtain milk powder.

### Milk powder production

Milk samples were concentrated in a vacuum evaporator at temperatures of 45–50 °C to reach their dry matter content of 45%. Then, the condensed milk samples were spray-dried using a Mini Spray Dryer B-290 (Buchi, Switzerland). A peristaltic pump was used to feed the samples to the atomization nozzle. The pump was working at 100% efficiency and 0.15% flow rate. To diversify technological parameters of milk powder production process, various temperatures of the inlet air were used during drying, i.e. 140, 150 and 160 °C.

The milk powders produced were transferred to hermetically closed containers and stored at room temperature until analyzed, but no longer than for 1 week.

### Scanning microscopy

The microstructure of milk powders was analyzed using a Quanta 200 scanning microscope (FEI Company, Eindhoven, the Netherlands). Powder samples were stuck with a carbon tape to stands, placed in a microscope chamber, and immediately frozen using a Peltier module at a temperature of − 18 °C. Observations were carried out at an accelerating voltage of 30 kV. A series of photographs were taken at magnifications of 400, 800, and 1600× for all samples of milk powders at randomly selected sites of the specimens. The goal was to obtain a high number of images of the particles of the analyzed milk powders to be used for successive image analysis.

### Image analysis coupled with determination of fractal dimension

Microstructural images of the particles of milk powders were analyzed with Image J software, developed at the National Institutes of Health (Bethesda, Maryland, USA) and available in the Internet. The images were opened in the software and transformed by scale setting and contrast increase using a “threshold” tool. Then, the perimeter and surface area of the particles were determined. The achieved series of dependencies between the perimeter and the surface area of the particles were used to determine the fractal dimension of their contour.

Using the following formula:

$${(A) \sim (P)}^{2/D}$$

where A—surface area, P—perimeter, and D—fractal dimension of the contour, straight lines with the slope of a = 2/D were obtained in logarithmic graphs log (A) = *f* (log (P)), which served to compute fractal dimensions of the particles of the analyzed milk powders (Dziuba et al. 1999).

Afterwards, particle diameters were computed from the values of particle surfaces and perimeters, assuming that the particles were spherical. These computations were used for a comparative analysis with results of particle size measurement with the laser diffraction method.

### Particle size determination with laser diffraction method

The particle size of the investigated milk powders was determined with the laser diffraction method in a Mastersizer 3000 instrument (Malvern, Malvern Instruments Ltd., Worcestershire, UK), with an Aero S attachment. During the measurement, the sample is dispersed through the acceleration of particles in a flux of compressed air in a Venturi’s nozzle. The material was fed from a feeder having a diameter of 3.5 mm at obscurance ranging from 0.1 to 50%. Volumetric diameters of particles, mean volumetric diameter D[4,3], and specific surface of the particles were determined. Measurements were conducted twice for each sample in five replications.

## Results and discussion

**Fig. 1:**
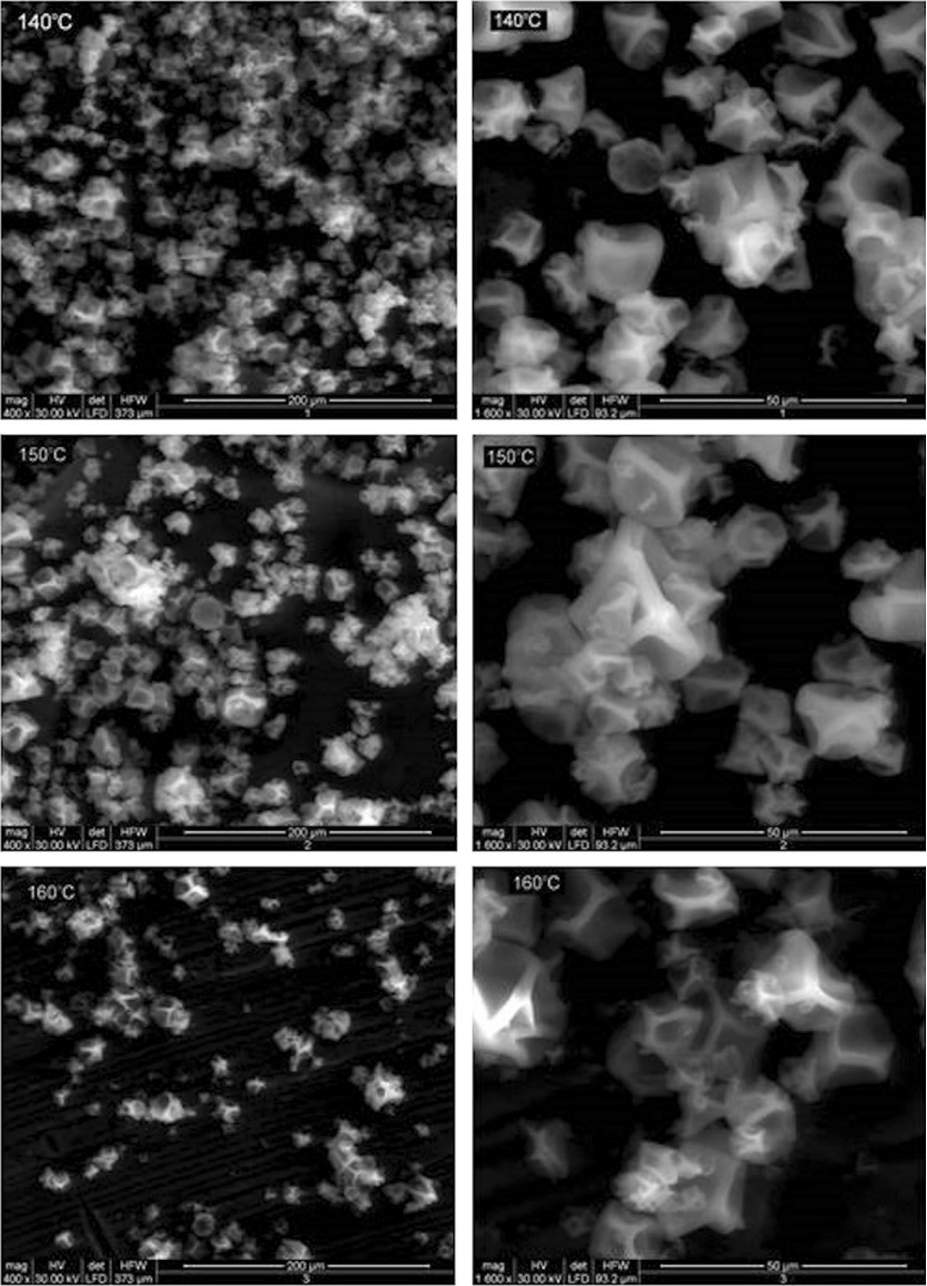
Images of the microstructure of milk powders (magnification: 400× and 1600×)

Skimmed milk with fat content of 0.26%, protein content of 2.96%, lactose content of 4.24%, and dry matter content of 8.24% was used in the study. To determine the effect of technological parameters on milk powders microstructure, various inlet air temperatures, i.e. 140, 150, and 160 °C, were used during milk drying as differentiating factors. The microstructure of the produced powders was observed in a scanning electron microscope (SEM). Microscope images of the powders are presented in Fig. 1.

As it appears from Fig. 1, the shape of powder particles was irregular, close to spherical, and numerous hollows could be observed on their surface. The microstructure of milk powders is significantly affected by the chemical composition of their surface. The development of hollow structures on their surface may be affected by a high protein content (Fyfe et al. 2011). Due to a low fat content in the studied samples, the impact of this component may be of lesser significance. The presence of lipids on particle surface impairs its wetting, while hydrophilic compound (lactose or minerals) reduce the wetting time (Burgain et al. 2017). This, in turn, affects the functional properties of dairy powders.

Observations of powders microstructure revealed the effect of drying temperature on their surface structure development. The higher temperature used in the study contributed to the slight smoothening of powder surface. At higher temperatures, the evaporation process proceeds faster, which makes the surface structure smoother and more regular. High temperatures may also contribute to enhanced denaturation of proteins, which affects protein structure and protein interactions with water, and ultimately impacts the structure of particles. However, the observation and comparison of the irregular microstructure, visible under the microscope, bears the risk of wrong and subjective assessment. Precise comparison of such structures may be achieved using image analysis and certain algorithms which enable detecting characteristic traits of the scrutinized objects. Hence, image analysis coupled with fractal dimension determination were used in this study in the evaluation of milk powder particles morphology.

**Fig. 2:**
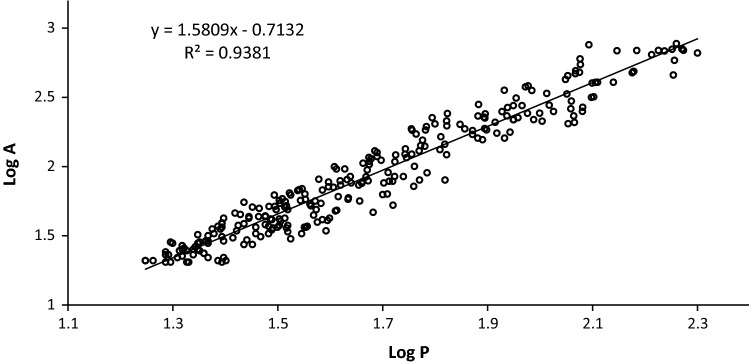
Dependency between the logarithm of perimeter and the logarithm of surface area of particles of milk powder produced at a temperature of 140 °C (A: area, P: perimeter)

The fractal dimension, determined from the dependency between the perimeter and surface area of a particle, indicates the extent of development of the surface of the analyzed particles and allows for precise and unbiased comparison of particle microstructures obtained at various drying temperatures. The fractal image analysis method has been used to analyze surfaces of particles or aggregates, but also to investigate crystallization properties of milk fat, yoghurt microstructure or properties of aggregates in wastewater (Staniewski et al. 2015; Smoczyński and Baranowska 2014; Smoczyński et al. 2016; Florio et al. 2019). Results of image analysis are presented in Table 1, whereas an example of a logarithmic dependency between particle perimeter and surface area is shown in Fig. 2.

**Table 1:** Results of image analysis with fractal dimensions determined for the analyzed samples of milk powders

Inlet temperature (°C)	Determination coefficient (R^2^)	Fractal dimension
140	0.94	1.26
150	0.93	1.21
160	0.90	1.20

The fractal dimension was determined based on at least 250 measurements of each sample of milk powder. High values of determination coefficients (R^2^) point to the good fit of the mathematical model to results obtained in the study.

Values of fractal dimensions determined in the study indicate small differences in the microstructure of milk powders. The fractal dimension was little higher for milk powder produced at 140 °C, which means that at the higher drying temperature the obtained surface of milk powder became smoother, and the contour became smoother and less ragged. This points to the effect of production process parameters on the microstructure and, by this means, on the properties of the milk powders. Even though the differences in fractal dimensions may be small, they still indicate some dependency between drying temperature and properties of milk powder microstructure, which may affect—to a lesser or greater extent—their functional properties. Importantly, the mathematical approach to microstructure evaluation allows for unequivocal detection of even subtle differences between the analyzed samples, which would not be possible via visual examination. Langrish et al. (2006) have determined fractal dimensions of particles produced using a laboratory-scale spray dryer using the laser light diffraction method. At higher drying temperatures, they achieved particles with only negligibly higher values of the fractal dimension, which were more collapsed and had higher bulk density. Nevertheless, the differences they observed were not large, despite a significant difference in inlet air temperatures, i.e. 120 and 200 °C.

**Fig. 3:**
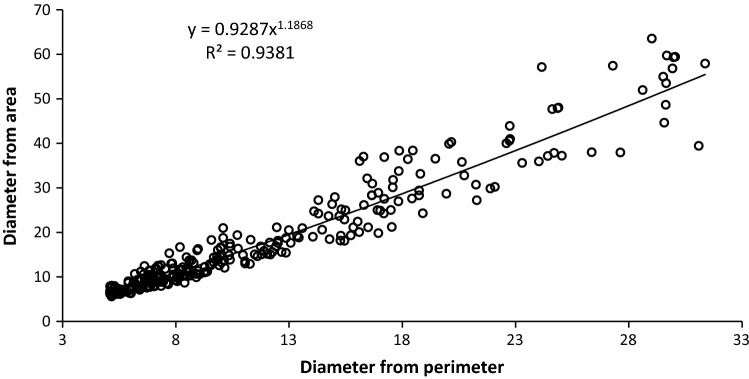
Dependency between diameters of particles of milk powders produced at 140 °C computed from particle perimeter and surface area

In this study, results of measurements of the perimeter and surface area of the analyzed particles were converted into diameters using formulas for circle perimeter (circumference) and circle area. Examples of the conversion were compared in Fig. 3.

Results presented in Fig. 3 together with the determination coefficient and curve equation confirm the advisability of using fractal dimension analysis in microstructure assessment. It may be seen from the figure that the dependency achieved is not linear. In the case of larger particles, differences in their diameter are greater because the greater the objects (magnification) are, the more details become noticeable on their surface, and this affects the outcome of particle perimeter measurement. This indicates the possibility of making mistakes in the assessment of powder particles microstructure at the assumption of the spherical shape of the powders. Similar dependencies were obtained for the other two samples of milk powders.

Afterwards, laser diffraction method was employed to determine particle size distribution. These determinations were carried out using the Mastersizer instrument with an Aero attachment for ‘dry’ analysis of powder particles. Table 2 presents results of these determinations, whereas an example of a logarithmic dependency between particle perimeter and surface area is shown in Fig. 2.

**Table 2:** Parameters of particle size distribution determined for milk powders produced at various temperatures

Inlet temperature (°C)	Dv10 (µm)	Dv50 (µm)	Dv90 (µm)	D[4,3] (µm)	Surface area (m^2^/kg)
140	7.29	17.00	31.05	18.25	490.45
150	8.33	18.97	34.23	20.27	436.63
160	6.73	17.40	32.95	18.80	617.65

The analysis of results indicates small differences in particle size distribution determined for the analyzed milk powders. Particle sizes at 140 °C are scattered and smaller in dimension. Slightly larger and coalesced particles were observed in the case of the milk powder produced at a drying temperature of 150 °C, which had the highest values of Dv10, Dv50, Dv90, and Sauter’s diameter D[4,3] (Fig. 1, Table 2). In turn, the largest specific surface was determined for the milk powder produced at 160 °C. This may be due to its smaller Dv10 and thus to a higher content of fine-size particles in this powder, which is responsible for the increase in surface area. As can be seen in Fig. 1 the powder particles formed at 160 °C are also slightly coalesced. A similar dependency was reported by Nikolova et al. (2014), which produced milk powders with smaller particles at higher drying temperatures. The lower value of fractal dimension determined for this powder, despite its large specific surface area, may point to differences in the structure of larger and smaller particles. The fractal dimension is an averaged value for all particles that may differ in structure. During drying at high temperature, the behavior of particles having small diameters may differ from the behavior of larger particles, hence the structure of particles with small and large diameters may differ as well. A significant role in the drying process is also ascribed to milk fat globules which at temperatures exceeding their melting point may migrate to the interior or remain on the surface of powder particles (Buma and Henstra 1970; Nijdam and Langrish 2005; Kim et al. 2003).

**Fig. 4:**
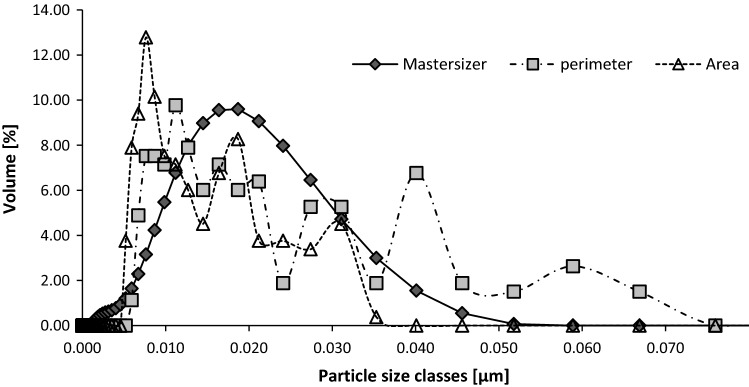
Particle size distribution determined for milk powder produced at 140 °C

Next, particle sizes achieved using the image analysis method were compared with these measured using the Mastersizer instrument. Figure 4 presents an exemplary particle size distribution for milk dried at 140 °C.

As seen in Fig. 4, sizes of particles computed from their perimeter differ from these computed from their surface area and attain higher values. This may be due to the irregular contour of the particles. Its elongation may cause a negligible increase in the diameter of the analyzed object. Considering the surface area measurement, results achieved indicate smaller sizes of the particles, because in the case of an irregular contour of a particle its real diameter would be greater than the diameter of a circle corresponding to this surface. The Mastersizer instrument measures laser light scattering on the analyzed particles; this measurement allows computing particle size distribution corresponding to this scattering. Results presented in the figure indicate that the particle sizes fit within the range of values determined in the image analysis, but represent some averaged values, whereas the course of the curve on the histogram is more regular and similar to the Gaussian curve. In spite of the fact that over 250 objects were examined during image analysis, the course of curves on the histogram is irregular. Similar results and a similar course of curves were obtained for the other samples of milk powder. In spite of the fact that the particle sizes determined with various methods fitted within the same range of values, the course of curves differed slightly, which shows differences in particle size distribution depending on the measurement method. This indicates the correctness and purposefulness of using the fractal dimension as a parameter enabling explicit and precise characterization of the morphological traits of milk powders.

## Conclusion

In the reported study, image analysis coupled with fractal dimension determination was used to characterize the microstructure of milk powder particles. Study results indicate that technological parameters used, like e.g. drying temperature, affect the microstructure of the milk powders produced. Values of fractal dimensions determined in the study decreased slightly along with drying temperature increase, which means that at the higher drying temperature the obtained contour of milk powder particles became smoother and less ragged. Apart from that, results of the image analysis of particle sizes were compared with corresponding results of instrumental analysis conducted with the Mastersizer instrument. Although particle sizes determined with both methods fitted within the same range of values, the contribution of particular fractions slightly differed. Each of the methods provides useful information and the choice of the appropriate approach may not always be unambiguous. To recapitulate, the fractal dimension used in this study provides explicit and precise characteristics of the microstructure of the analyzed milk powders. Therefore it can be used to study irregular structures and the impact of technological processes on the microstructure of different food products.
